# Risk association model for atelectasis complication in *Mycoplasma pneumoniae* pneumonia patients following standardized treatment

**DOI:** 10.3389/fped.2024.1422074

**Published:** 2024-11-28

**Authors:** Mingyi Xu, Minhao Fan, Huixia Wang, Jun Qian, Yi Jiang, Yifan Zhu, Deyu Zhao, Feng Liu, Yun Guo, Ling Li

**Affiliations:** ^1^ Department of Respiratory Medicine, The Affiliated Wuxi People's Hospital, Wuxi Children's Hospital of Nanjing Medical University, Wuxi, Jiangsu, China; ^2^ Department of Respiratory Medicine & Clinical Allergy Center, Affiliated Children's Hospital of Jiangnan University (Wuxi Children's Hospital), Wuxi, China; ^3^ Department of Respiratory Medicine, Zhumadian Central Hospital, Zhumadian, Henan, China; ^4^ Department of Respiratory Medicine, Children’s Hospital of Nanjing Medical University, Nanjing, China

**Keywords:** children, *M. pneumoniae*, *M. pneumoniae pneumonia*, atelectasis, association model

## Abstract

**Background:**

*Mycoplasma pneumoniae* pneumonia (MPP) is a common disease of childhood pneumonia, and atelectasis is a serious comorbidity. Traditional diagnostic methods for MPP are limited by low accuracy, emphasizing the need for improved diagnostic approaches. This study aimed to establish a predictive scoring model for early detection of MPP complicated with atelectasis following standardized treatment.

**Methods:**

A total of 572 children were retrospectively enrolled, including 40 patients with MPP complicated by atelectasis despite standardized treatment and 532 patients in the non-atelectasis group. Clinical, laboratory, and imaging data within 24 h of admission were collected, including demographic information and various biomarkers. Multivariate logistic regression analysis was employed to identify risk factors and construct a predictive model, evaluated using receiver operating characteristic (ROC) curve analysis.

**Results:**

Significant differences were observed between the MPP complicated with atelectasis group and the non-atelectasis group in terms of age, hospital admission time, fever duration, neutrophil percentage and count, CRP, ALT, and LDH levels (*P* < 0.05). According to the multivariate logistic regression analysis, length of fever, neutrophil ratio, platelet count, ALT, LDH, age were incorporated into the nomogram. The predictive model exhibited a sensitivity of 87.97% and specificity of 77.50% according to the ROC curve.

**Conclusion:**

Our study presents a preliminary risk association model incorporating clinical indicators such as fever duration, neutrophil ratio, platelet count, ALT value, LDH value, and age to aid in the early prediction of atelectasis in children with MPP. Given the methodological limitations, the generalizability of our findings is constrained, and this model should be viewed as an initial framework for clinical assessment rather than a definitive tool.

## Introduction

*Mycoplasma pneumoniae* (MP) is the smallest microorganism known to survive independently. Among children aged approximately 5 years, Mycoplasma pneumoniae pneumonia (MPP) constitutes the most prevalent form of community-acquired pneumonia (CAP) (1, 2). MP infection is prevalent worldwide, accounting for 15%–50% of CAP pathogens. School-age children are particularly susceptible to infections. The epidemic cycle is generally 4–7 years, and the infection rate can be as high as 30%–50% (3–6). However, in 2023, a global outbreak of MP infection occurred, with China experiencing a significant surge, reaching an infection rate as high as 61.1% (7, 8). This outbreak led to a large number of MPP cases accompanied by complications. Children with severe or refractory MPP can develop pleural effusion, atelectasis, or necrotizing pneumonia. Some children become critically ill and die (9–11). Furthermore, severe MP infection can also leave sequelae such as chronic pulmonary interstitial fibrosis, bronchiolitis obliterans, unilateral pulmonary abnormal light syndrome, and reduced lung diffusion function, seriously affecting children's physical and mental health and increasing family and social burdens (12, 13).

Atelectasis represents a severe pulmonary complication associated with MPP, potentially affecting one or more pulmonary segments and reducing lung volume or air content (14). Regarding clinical features, children with MPP complicated with atelectasis often have higher C-reactive protein, neutrophil-to-lymphocyte ratio, and LDH lactate dehydrogenase levels and more complications, including erythema, liver damage, pleural effusion, and hypercoagulability. Regarding clinical outcomes, children with MPP complicated with atelectasis have a higher proportion of refractory cases, ICU admissions, and patients undergoing oxygen therapy; significantly longer total fever duration and hospital stay; greater hospital expenses; and are more prone to necrotizing pneumonia (15). These complications make atelectasis clinically intractable. Despite treatment, some children with atelectasis persist, and lung fibrosis was observed with concomitant local repeated infections, necessitating lung resection intervention (16, 17).

Therefore, early detection of atelectasis in children with MPP and the implementation of proactive treatment measures based on early prediction to mitigate pulmonary inflammation progression are crucial. Identifying and diagnosing MPP complicated by atelectasis in its early stages is challenging, especially for children undergoing standardized treatment, as physicians may not frequently perform imaging examinations, leading to underdiagnosis of atelectasis. By the time it is discovered in later stages, the optimal treatment window may have been missed. Traditional diagnosis heavily relies on physician judgment, which can be subjective and influenced by clinical experience and diagnostic biases. Establishing straightforward, sensitive, and specific diagnostic scoring scale is therefore paramount for optimizing clinical diagnosis, tailoring treatment, and assessing potential adverse prognostic risks. In this study, we retrospectively analyzed the clinical characteristics and laboratory and imaging data of children with MPP who developed atelectasis despite standardized treatment, alongside a non-atelectasis group. Additionally, we developed a nomogram association model to forecast the occurrence of atelectasis in children with MPP, aiming to facilitate early diagnosis and intervention for this comorbidity.

## Materials and methods

### Ethics approval and informed consent

This study was approved by the Medical Ethics Committee of Wuxi Children's Hospital (no. WXCH2020-02-005) and registered in the Chinese Clinical Trial Registry (registration number: ChiCTR2000038742). All the methods used in this study were conducted following the Declaration of Helsinki. All children enrolled in the study provided informed consent from their parents or legal guardians.

### Study population

The inclusion criteria were as follows: 572 children hospitalized for the first time in the Department of Respiratory Medicine of Wuxi Children's Hospital or Nanjing Children's Hospital were included. All children met the diagnostic criteria of the MPP guidelines (18). The clinical manifestations included fever, irritating dry cough, shortness of breath, and other systemic manifestations. Among the 572 children included in the study, 287 underwent only chest x-ray, 33 underwent only chest CT scans, and 252 underwent both chest x-ray and chest CT scans (including chest CT done on an outpatient basis or in other hospitals), and chest x-ray or chest CT findings suggestive of inflammatory infiltrates. The etiological test showed that the MP-immunoglobulin M (IgM) was positive, the antibody titer was more than 1:160, or the polymerase chain reaction result for *M. pneumoniae* is positive (18, 19). The diagnosis of atelectasis was based on imaging manifestations included increased opacity of affected lobe, bronchovascular crowding, narrowing of the ipsilateral intercostal spaces, compensatory hyperinflation, compensatory shift of adjoining structures and/or diaphragm, hilar displacement, ipsilateral hemithoracic contraction (in massive collapse), and silhouette sign affecting contiguous mediastinal structures (20). Exclusion criteria: 1. pulmonary infection with other pathogens or chronic pulmonary inflammation; 2. basic diseases or malignant tumors of the liver, kidney, heart, brain, and other systems; 3. without informed consent or incomplete data collection.

The group was divided into an Atelectasis group and a Control group according to the presence or absence of radiographic signs of atelectasis following 7 days of conventional treatment. This treatment regimen included macrolide antibiotics, corticosteroids, expectorants, and various other symptomatic therapies.

### Data collection

We collected the following common variables for hospitalized children, including the following: 1. Continuous variables: age, hospitalization days, fever days before admission, blood routine within 24 h of admission: white blood cell count (WBC), neutrophil percentage, hemoglobin (HB), platelet count (PLT), C-reactive protein (CRP), alanine aminotransferase (ALT), aspartate aminotransferase (AST), lactate dehydrogenase (LDH), creatine kinase isoenzyme (CK -MB); 2. Binary categorical variables: sex, presence or absence of pleural effusion at admission, whether there was large-area lung consolidation on chest imaging, and whether there were related underlying diseases; 3. Nominal variables: antibodies against common respiratory tract pathogens, serum mycoplasma antibody titer, blood culture, sputum culture, and alveolar lavage fluid culture.

### Statistical analysis

R × 64 3.6.0 and RStudio for Windows (Auckland, New Zealand) were used for data processing. Statistical significance was set at *P* < 0.05. The *t*-test was used for normally distributed continuous data, and the median and interquartile range (IQR) for non-normally distributed data were calculated. Categorical variables were analyzed using the chi-squared test. A multivariate analysis was performed using a logistic regression model to determine the risk factors related to the occurrence of atelectasis complication in MPP patients following standardized treatment. The final regression model was transformed into a nomogram using the R software. Taking sensitivity as the ordinate and 1-specificity as the abscissa, a receiver operating characteristic (ROC) curve was drawn, the regression model of atelectasis MPP was predicted, and the specificity and sensitivity of the prediction scale were calculated.

## Results

**Figure 1:**
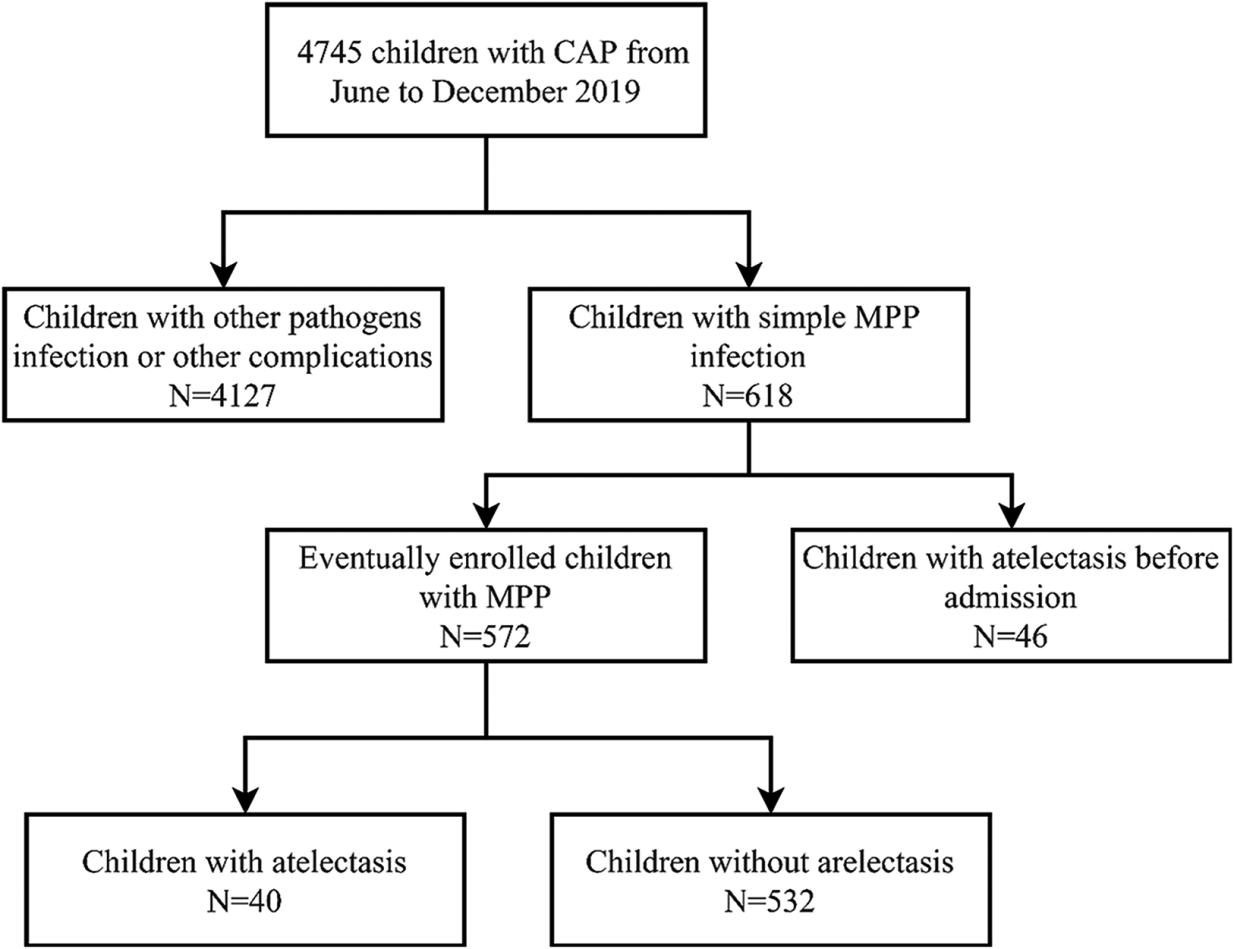
Research flow chart: patient recruitment and exclusion. CAP, community-acquired pneumonia; MPP, *M. pneumoniae* pneumonia.

The research group retrospectively evaluated 4,745 children with pneumonia admitted to Wuxi Children's Hospital and Nanjing Children's Hospital from June to December 2020. After statistical analysis, 4,127 cases of infection with other pathogens, co-infections, or co-extrapulmonary diseases were excluded. Additionally, 618 cases of mycoplasma pneumonia consistent with simple mycoplasma pneumonia, of which 46 cases had atelectasis on admission and were excluded. Finally, 572 children were included in the MPP group, and the children were classified into atelectasis and non-atelectasis groups according to lung imaging findings after 7 days of conventional treatment, which included macrolide antibiotics, corticosteroids, expectorants, and other symptomatic treatments. Among them, 40 cases were complicated with atelectasis after treatment, while 532 cases were not (Figure 1).

A total of 572 patients with confirmed MPP were included in this study. The clinical, laboratory, and imaging data of children with MPP were collected within 24 h of admission, and additional imaging data were collected after 7 days of conventional treatment. The clinical features, laboratory findings, and imaging findings of the two groups were compared.

There was no statistically significant difference in sex distribution between the atelectasis and non-atelectasis groups. However, the mean age of onset in the atelectasis group was significantly higher than in the non-atelectasis group (5.25 vs. 3.17, *P* < 0.05). Additionally, the length of hospital days (13.00 vs. 8.00, *P* < 0.05) and the number of days with fever (8.00 vs. 4.00, *P* < 0.05) were significantly higher in the atelectasis group than in the non-atelectasis group.

Compared to the non-atelectasis group, the percentage of neutrophils (67.09% vs. 56.35%, *P* < 0.05), neutrophil count (6.24 × 10^9^ vs. 4.52 × 10^9^, *P* < 0.05), CRP levels (24.00 ng/ml vs. 8.00 ng/ml, *P* < 0.05), ALT levels (34.00 U/L vs. 15.00 U/L, *P* < 0.05), and LDH levels (592.00 U/L vs. 318.50 U/L, *P* < 0.05) in the atelectasis group were significantly increased.

Furthermore, the two groups had no statistically significant differences in the other laboratory results (Table 1). The presence or absence of a pleural response was not statistically different between the two groups (7.1% vs. 7.3%, *P* > 0.05); however, imaging results showed more pleural effusion in the atelectasis group (36% vs. 5%, *P* < 0.05) (Table 2).

**Table 1:** Admission information of children with Mycoplasma pneumoniae pneumonia and atelectasis and control group.

Variables	Atelectasis (*n* = 40)	Control group (*n* = 532)	c^2^/Z	*P* value
Male (%)	24 (57.14%)	295 (55.45%)	0.045	0.482
Age (year,)	5.25 (4.00–7.96)	3.17 (1.83–5.50)	4.430	0.000
length of stay (days)	13.00 (9.00–16.00)	8.00 (7.00–10.00)	−4.785	0.000
length of fever (days)	8.00 (7.00–9.50)	4.00 (2.00–6.00)	−6.149	0.000
WBC (×10^9^/L)	9.03 (6.56–11.54)	8.52 (6.80–11.11)	−0.948	0.343
Neutrophils (%)	67.0900 (63.3450–78.900)	56.3500 (43.5000–66.0900)	−4.138	0.000
Neutrophil count (×10^9^/L)	6.24 (3.44–9.24)	4.52 (3.09–6.51)	−2.818	0.005
CRP (mg/L)	24.00 (8.00–52.00)	8.00 (8.00–15.00)	−2.508	0.012
Hb (g/L)	122.00 (114.50–129.00)	123.00 (117.00–130.00)	−1.546	0.122
PLT (×10^9^/L)	265.00 (216.50–296.00)	253.00 (197.00–324.00)	−1.691	0.091
AST (U/L)	37.00 (20.00–59.00)	30.00 (24.00–39.00)	−0.893	0.372
ALT (U/L)	34.00 (19.50–59.00)	15.00（11.00–21.00)	−7.041	0.000
LDH (U/L)	592.00 (285.50–771.50)	318.50 (270.00–405.00)	−3.126	0.002
CK-MB (U/L)	20.00 (17.00–29.50)	22.00 (18.00–32.00)	−0.036	0.971

The values are spaced at ±25% of the median. WBC, white blood cell count; CRP, C-reactive protein; HB, hemoglobin; PLT, platelets; AST, aspartate aminotransferase; ALT, alanine aminotransferase; LDH, lactate dehydrogenase; CK, creatine kinase; CK-MB, creatine kinase isoenzyme.

**Table 2:** Chest symptoms.

Variables	Atelectasis(*n* = 40)	Control group (*n* = 532)	c^2^/Z	*P* value
Pleural reaction (%)	0.071	0.073	0.121	0.730
Pleural effusion(%)	0.36	0.05	59.85	0.000

Multivariate logistic regression analysis was conducted to determine the weight of each index in predicting the occurrence of atelectasis. The weights of the indices, listed in descending order, were as follows: duration of fever, ALT levels, neutrophil percentage, LDH levels, PLT, and age. Duration of fever, ALT levels, neutrophil percentage, LDH levels, PLT were independent factors influencing the occurrence of atelectasis in MPP patients following standardized treatment (Table 3).

**Table 3:** Multivariate logistic regression analysis of the weight of each value.

	Estimate	Std.Error	*Z* value	Pr(>|Z|)
length of fever (days)	2.388 × 10^–1^	5.247 × 10^–2^	4.550	5.36 × 10^–6^***
N.(%)	4.156 × 10^–2^	1.617 × 10^–2^	2.570	0.01016*
PLT(×10^9^/L)	3.879 × 10^–3^	1.800 × 10^–3^	2.155	0.03116*
ALT (U/L)	2.385 × 10^–2^	7.929 × 10^–3^	3.007	0.00264**
LDH (U/L)	1.858 × 10^–3^	8.537 × 10^–4^	2.177	0.02949*
Age (year)	1.225 × 10^–1^	7.513 × 10^–2^	1.631	0.10285

N, neutrophil percentage; PLT, platelets; ALT, alanine aminotransferase; LDH, lactate dehydrogenase.

*P* < 0.05 was considered statistically significant, *P < 0.05, **P < 0.01, ***P < 0.001.

Variables including days of fever, neutrophil percentage, ALT, LDH values, and PLT were statistically significant. Although children's age did not exhibit significant differences in the multivariate analysis, it had been significant in previous studies (21, 22). The nomogram assigns a weighted score to each independent risk factor, with a maximum of 100 points per factor. The overall risk of atelectasis, ranging from 0.001 to 0.95, is calculated by summing the scores for all high-risk factors. A higher cumulative score corresponds to an increased likelihood of atelectasis occurrence.

**Figure 2:**
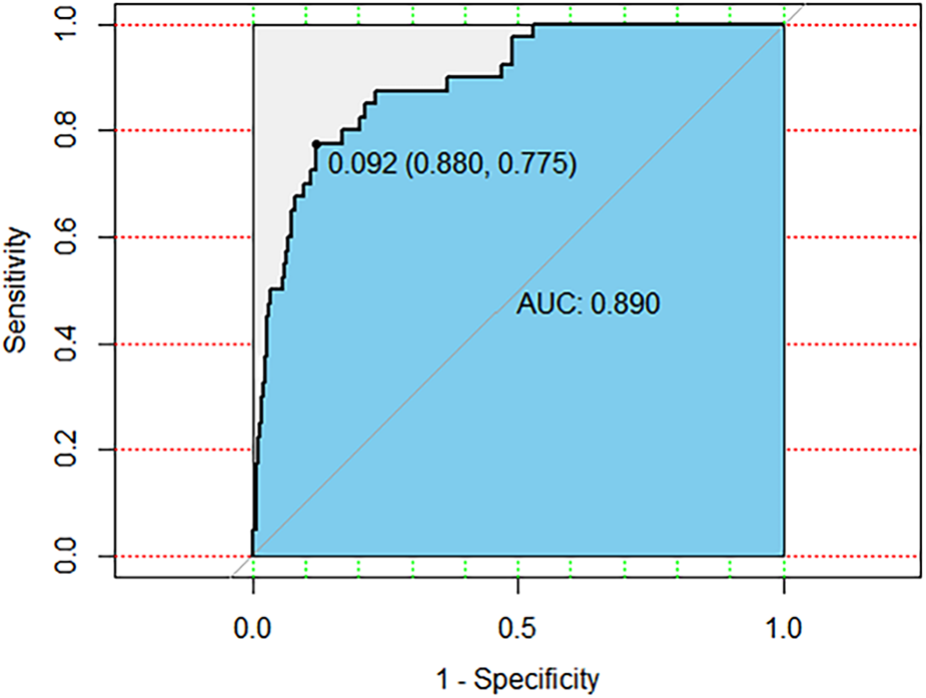
The ROC curve is drawn based on the clinical prediction model of MPP with atelectasis. The AUC is 0.890, the most the best critical probability is 0.092.

ROC curves were drawn to determine the predictive model based on the best critical probability using a confusion matrix to validate the model internally (Figure 2). The sensitivity and specificity of the evaluation model were 87.97% and 77.50%, respectively, and the model correctly classified 87.24% of the study subjects (Table 4). These results suggested that the early association model can be used for the early prediction of atelectasis in children with MPP.

**Table 4:** MPP combined with atelectasis prediction model.

		Observed		Percent of total
		Without atelectasis	With atelectasis	
Predicted	Without atelectasis	468	9	98.11%
	atelectasis	64	31	32.63%
Percentage correction		87.97%	77.50%	87.24%

The sensitivity is 87.97%, the specificity is 77.50%, the negative predictive value is 98.11%, the positive predictive value is 32.63%, and 87.24% of the subjects can be correctly classified.

**Figure 3:**
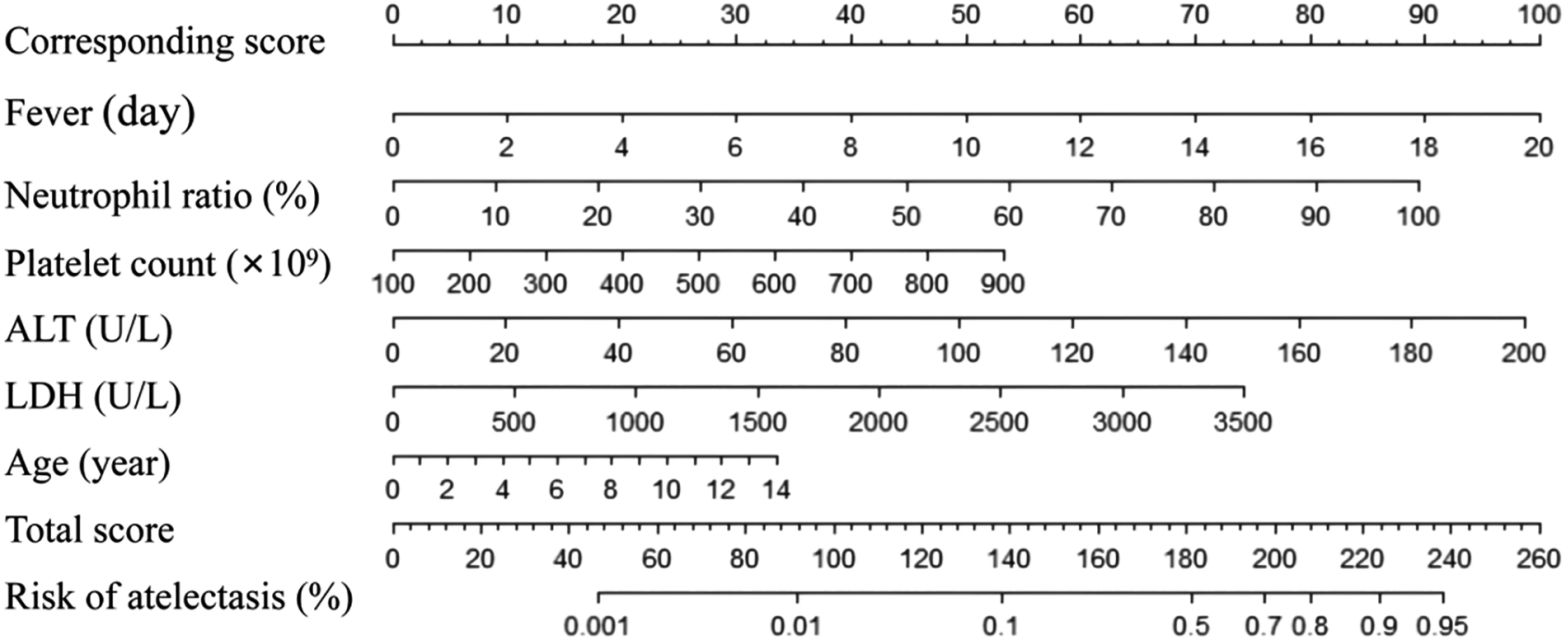
Nomogram MPP atelectasis clinical prediction assessment scale. The score in the first row corresponds to the value of each of the following indicator, and the score is calculated based on the value of each variable. Evaluate the risk probability of MPP atelectasis based on the total score. How to use the table: Enlarge and print it on A4 paper according to the scale, use a ruler and other measuring tools to calculate the corresponding score.

To facilitate clinical application, a nomogram based on the logistic regression model was developed, enabling clinicians to quickly calculate a risk score based on clinical symptoms and laboratory tests. The nomogram allows for the assessment of atelectasis risk scores based on the following metrics (Figure 3).

## Discussion

MP is a common pathogen associated with respiratory tract infections in children. MP infection often cause multi-system damage inside and outside the lung through cell adhesion, toxin invasion, and excessive immune damage (5, 6, 23–27). Recently, the number of children with severe or refractory MPP appears to have increased with the emergence of drug resistance. This increase may be accompanied by a rise in complications such as atelectasis, pulmonary fibrosis, and bronchiolitis obliterans, which potentially pose serious threats to the safety and quality of life of affected children. Therefore, despite the critical need for early identification of severe or refractory MPP (2, 28, 29), there remains a lack of comprehensive risk association models, particularly for MPP complicated with atelectasis. This gap in knowledge makes it challenging for clinical pediatricians to effectively guide patient management strategies.

The radiographic signs of atelectasis and lung consolidation are different. Direct signs of atelectasis on chest films include fissure deviation, parenchymal opacification with unbroken linear borders, and vascular displacement (20). Pulmonary consolidation refers to the replacement of alveolar air by pathological fluids, cells, or tissues and manifests an increase in the density of the lung parenchyma, resulting in obscuring the margins of vessels and airway walls (30). Additionally, the air bronchogram may be present.

To predict the risk of MPP complicated with atelectasis and potentially reduce the occurrence of atelectasis complications, we have identified six indicators, including fever days, neutrophil percentage, PLT, ALT, LDH, and age, as predictors of pulmonary atelectasis. In this study, the number of days of fever was analyzed, and it was observed that the fever duration in the MPP combined with the atelectasis group was longer than that in the non-atelectasis group. Jang (31) suggested that fever is a risk factor for bronchial mucus obstruction in children with MPP, which has also been reported previously (32). The neutrophil percentage is a common clinical indicator of infection. Several studies suggest that the extracellular traps of neutrophils may play a crucial bactericidal role in infectious diseases such as CAP (33, 34). An increase in the proportion of neutrophils indicates that the infection is worsening (14, 35). Furthermore, it's noteworthy that these indicators were observed in children who still developed atelectasis despite standardized treatment protocols. This underscores the importance of early recognition and intervention strategies even in cases where conventional treatment has been administered.

Some studies suggest that MP infection can potentially lead to the release of several inflammatory factors, resulting in endothelial cell damage and platelet aggregation, leading to a hypercoagulable state and thrombosis (36). Furthermore, Ling (37) reported the severe clinical status of patients with lower respiratory tract infections and thrombocytosis. This suggests a potential link between MP infection and thromboembolic events, although further research is needed to elucidate the underlying mechanisms.

Additionally, elevated levels of ALT and LDH, as observed in our study, may indicate extrapulmonary damage associated with MP infection. Previous studies have reported that MP infection can cause *M. pneumonia*-associated hepatitis and elevated liver enzymes (38, 39). However, elevated LDH levels are thought to reflect generalized tissue damage or cellular necrosis. Previous studies have also suggested a potential association between elevated LDH levels and epithelial damage in the lungs (40, 41), these findings underscore the systemic impact of MP infection and highlight the importance of monitoring LDH levels as a marker of tissue injury in affected patients. Importantly, our study further demonstrates the significance of LDH in the early identification of atelectasis complicating MPP.

In this retrospective study, we found that the median age at onset in the MPP combined with atelectasis group was higher than in the non-atelectasis group. Studies have shown that the main pathogenic mechanism of MPP is related to an excessive immune response in the body, which in turn is related to the relative immaturity of the immune mechanism in young children. Excessive inflammation tends not to be produced in young children; however, the opposite is true in older children. This may be one of the causes of MPP complicated with atelectasis in older children (26, 42, 43). While excessive inflammation is less commonly observed in young children, the opposite tends to be true in older children. This age-related difference in immune response may contribute to the increased risk of developing atelectasis complicating MPP in older children.

There have been previous studies on predictive scales related to MPP and refractory MPP and studies on MPP and the risk prediction model of bronchiolitis obliterans (22, 44). However, there are no studies on the risk model of MPP combined with atelectasis. Risk association models incorporate fever duration, neutrophil ratio, PLT, ALT, LDH, and age into clinical association models.

The ROC curve was drawn for each predictor; the area under the curve (AUC) was 0.890, and the optimal critical probability was 0.092, suggesting high specificity and sensitivity. These results demonstrate a high level of specificity and sensitivity in predicting the risk of MPP complicated with atelectasis.

An increasing body of evidence suggest that an excessive host immune response plays an important role in the pathogenesis of atelectasis (37, 45, 46). As a result, early administration of glucocorticoids may be a promising treatment option for children with MPP complicated by atelectasis (45).

This predictive model potentially offers several significant advantages. Firstly, it could enhances the ability of healthcare providers to promptly recognize and stratify patients at high risk of developing atelectasis, allowing for timely and targeted interventions to mitigate potential complications and improve patient outcomes. Secondly, the model's simplicity and ease of use might make it suitable for implementation across a wide range of healthcare settings, from primary care clinics to tertiary hospitals, facilitating its widespread adoption and integration into routine clinical practice. Additionally, the high specificity and sensitivity of the model, as indicated by the robust performance metrics such as the area under the ROC curve, underscore its reliability and effectiveness in accurately predicting the risk of atelectasis in children with MPP.

By potentially enabling early identification and intervention, our predictive model may significantly reduce the morbidity and mortality associated with MPP complicated by atelectasis, thereby alleviating the burden on healthcare systems and improving the overall quality of care for pediatric patients with respiratory infections. Furthermore, as the incidence of MPP continues to rise globally, particularly in regions experiencing outbreaks such as China, the need for effective risk association models to guide clinical management becomes increasingly imperative. In this context, our study represents a crucial step forward in advancing our understanding of MPP-related complications and improving clinical outcomes for affected children.

However, our study has several limitations that should be acknowledged. Firstly, as a retrospective study, it lacks prospective data collection and external validation. Therefore, the utility and generalizability of our experimental findings and nomogram require validation across multiple centers. Additionally, the relatively small sample size of patients with MPP and atelectasis in our study may limit the robustness and generalizability, which may limit the robustness and generalizability of our findings. As such, further data collection and external multicenter validation are warranted to confirm the reliability and applicability of our predictive model in diverse clinical settings.

## Conclusion

In conclusion, while our study provides a preliminary risk association model incorporating clinical indicators such as fever duration, neutrophil ratio, platelet count, ALT value, LDH value, and age to support early prediction of atelectasis in children with MPP, we acknowledge that methodological constraints limit the generalizability of our findings. As a retrospective analysis with a relatively small sample size and limited external validation, this model should be interpreted with caution and further validated through prospective, multicenter studies. The proposed model, therefore, serves as an initial framework for clinical assessment, rather than a definitive tool, underscoring the need for additional research to refine and confirm these predictive associations in diverse clinical settings.

## Data Availability

The original contributions presented in the study are included in the article/Supplementary Material, further inquiries can be directed to the corresponding authors.
